# Brain mediators of systemic oxidative stress on perceptual impairments in Parkinson’s disease

**DOI:** 10.1186/s12967-015-0749-9

**Published:** 2015-12-21

**Authors:** Wei-Che Lin, Kun-Hsien Chou, Pei-Lin Lee, Yung-Cheng Huang, Nai-Wen Tsai, Hsiu-Ling Chen, Kuei-Yueh Cheng, Hung-Chen Wang, Tsu-Kung Lin, Shau-Hsuan Li, Meng-Hsiang Chen, Cheng-Hsien Lu, Ching-Po Lin

**Affiliations:** Department of Diagnostic Radiology, Kaohsiung Chang Gung Memorial Hospital, Chang Gung University College of Medicine, Kaohsiung, Taiwan; Brain Research Center, National Yang-Ming University, Taipei, Taiwan; Department of Biomedical Imaging and Radiological Sciences, Institute of Neuroscience, National Yang-Ming University, 155 Li-Nong St., Sec. 2, Peitou, Taipei, Taiwan; Department of Nuclear Medicine, Kaohsiung Chang Gung Memorial Hospital, Chang Gung University College of Medicine, Kaohsiung, Taiwan; Department of Neurology, Kaohsiung Chang Gung Memorial Hospital, Chang Gung University College of Medicine, 123, Ta Pei Road, Niao Sung, Kaohsiung, Taiwan; Department of Neurosurgery, Kaohsiung Chang Gung Memorial Hospital, Chang Gung University College of Medicine, Kaohsiung, Taiwan; Department of Internal Medicine, Kaohsiung Chang Gung Memorial Hospital, Chang Gung University College of Medicine, Kaohsiung, Taiwan

**Keywords:** MRI, Mediation, Oxidative stress, Parkinson’s disease, Perceptual function

## Abstract

**Background:**

Parkinson’s disease (PD) is well documented to be associated with elevated systemic oxidative stress and perceptual impairments. Furthermore, the striatum and extrastriatal cortical areas, which are involved in the coordination of perceptual functions, are impaired at an early stage of the disease. However, the possible pathophysiology involved in perceptual impairments remains unclear. This raises the possibility that structural abnormalities might mediate the relationship between oxidative stress and perceptual impairments.

**Methods:**

We explored the differences between 27 patients with PD and 25 healthy controls in terms of serum oxidative stress, perceptual functions, and regional gray matter. A single-level three-variable mediation model was used to investigate the possible relationships between serum oxidative stress, regional gray matter volume, and different domains of perceptual functioning.

**Results:**

The results demonstrate that increased serum oxidative stress (as indicated by thiobarbituric acid reactive substances) was associated with declined perceptual functioning in PD patients. We further explored significant gray matter volume reductions in the bilateral temporal gyri (middle temporal gyrus and fusiform gyrus), bilateral frontal gyri, limbic lobe (hippocampus and uncus), left inferior parietal lobule, right caudate nucleus, and insula in PD. Further mediation analysis showed that gray matter volumes in the middle temporal gyrus, inferior parietal lobule, hippocampus, and insula served as brain mediators between elevated serum oxidative stress and perceptual impairments.

**Conclusions:**

These results suggest that higher oxidative stress levels adversely impact perceptual functions by causing temporal and mesolimbic abnormalities.

**Electronic supplementary material:**

The online version of this article (doi:10.1186/s12967-015-0749-9) contains supplementary material, which is available to authorized users.

## Background

The patterns of cognitive impairment in Parkinson’s disease (PD) are increasingly seen as being of clinical significance. In addition, specific cognitive domains may not only have different regional underpinnings but may also differ in their pathologic substrates [1, 2]. In PD, the presence of perceptual difficulties is common [3]. More specifically, these perceptual deficits may be unique among the cognitive impairments found in PD and are distinct from the effects of generalized dementia in several regards [4, 5]. This raises the possibility that the pathophysiology and vulnerable regions involved in the perceptual impairments of patients with PD might be independent of the motor and cognitive statuses of such patients. An understanding of the neuronal substrates underlying the deficits in perceptual functioning is essential to the development of targeted therapeutic strategies.

Studies of causative PD genes have confirmed the involvement of oxidative stress and apoptosis in the degradation of dopaminergic neurons in PD [6]. A recent PET imaging study demonstrated that striatal oxidative stress is enhanced in PD patients compared with controls and that this stress increases with the progression of disease severity, particularly in the contralateral striatum [7]. In addition, increased systemic oxidative stress has also been considered to induce ongoing CNS inflammation and dopaminergic neuronal death [8]. The production of highly toxic free radicals, which suggests increased oxidative stress, has been positively correlated with spatial memory deficits in a rat PD model [9] and could be protected against through the inhibition of oxidative stress [10]. These findings suggest a critical role of increased oxidative stress on cognitive deficits in PD. Unfortunately, research into the potential neural loss mechanisms associated with the severity of cognitive impairment, especially with the impaired perceptual functioning in PD patients, has thus far been limited [11].

Although markedly elevated neuroinflammation in patients with idiopathic PD has been found in the striatum, as well as in extrastriatal cortices, such as the frontal and temporal cortical regions, compared to age-matched healthy controls [12–14], it is still not known whether or not the increased systemic oxidative stress might directly or indirectly affect those vulnerable regions causing perceptual function impairment. In community-dwelling healthy elderly subjects, elevated serum high-sensitivity C-reactive protein has been thought to decrease regional gray matter volume in the posterior and lateral aspects of the left temporal cortex [15]. Damage initiated either from the CNS or by systemic oxidative stress may be propagatively transferred to the temporal regions directly or indirectly in patients with PD [16]. Furthermore, temporal regions have been reported to be the most susceptible to direct pro-inflammatory cytokine injection [17]. It is reasonable to infer that both elevated oxidative stress and decreased extra-striatal gray matter volume or the interaction between them might affect perceptual performance in PD, but this inference has not been tested until now.

The increased lipid peroxidation product, in terms of the level of thiobarbituric acid reactive substances (TBARS), along with the decreased level of endogenous antioxidant molecules, such as thiol in the brain, could contribute to dopaminergic neuronal death [18]. To gain a better understanding of the relationship between these different factors, we conducted a study examining the associations between the systemic TBARS and thiol levels, the structural volumetric morphology of the brain, and the performance intelligence quotient (IQ) in patients with PD and healthy control subjects. Our hypotheses were as follows: (1) Compared to healthy participants, PD participants will have increased TBARS and reduced thiol serum levels, and also impaired perceptual functions; (2) The increased systemic oxidative stress will be related to decreased perceptual functions; (3) Regional gray matter volume reductions will be found in PD subjects; and (4) Gray matter volume in those vulnerable anatomies will mediate the relationship between oxidative stress level and perceptual functioning. According to these hypotheses, peripheral oxidative stress might serve as the instigator that impacts the anatomical structures of those most susceptible regions and thus leads, in turn, to a decline in perceptual functioning.

## Methods

### Participants

The study was approved by the Local Ethics Committee on Human Research of Kaohsiung Chang Gung Memorial Hospital in Taiwan. All participants or their guardians provided written informed consent prior to participation in the study. Twenty-seven right-handed PD patients (11 men and 16 women, mean age: 54.6 ± 9.3 years) with no previous history of neurological or psychiatric illnesses, psychotropic medication, or contraindication to magnetic resonance imaging (MRI) were prospectively enrolled at the Neurology Department of Kaohsiung Chang Gung Memorial Hospital. Patients were included if they had been diagnosed with idiopathic PD by an experienced neurologist according to the Parkinson’s Disease Society’s criteria for idiopathic Parkinson’s disease [19]. Of the 28 PD patients, ten had never used any anti-Parkinson’s medication, whereas the others used dopaminergic medication (levodopa and dopamine agonists). The disease severity and functional status of each patient were evaluated with the Unified Parkinson’s Disease Rating Scale (UPDRS) [20], the modified Hoehn and Yahr stages (HY-stage) [21], and the Schwab and England activities of daily living scale (SE-ADL) [22] in the “OFF” state, which refers to when levodopa appears to become less effective in eliminating motor symptoms and the patients are unable to function properly. The patients’ mean disease duration, defined as the time since the given patient subjectively noticed his or her first symptoms, was 4.1 ± 3.7 years. For comparison, 25 sex- and age-matched healthy subjects (11 men and 14 women, mean age: 50.9 ± 10.5 years) with no medical history of neurologic diseases or psychiatric illnesses, alcohol/substance abuse, or head injury, and with similar levels of education, were recruited.

### Laboratory measurements for oxidative stress factors

We evaluated the oxidative stress condition in all subjects by measuring the serum concentration of TBARS and thiol. All subjects received blood sampling on the same day as the MRI study and neuro-psychological testing. Sera were isolated from peripheral blood samples drawn from each subject before and after the examination.

The serum TBARS were measured based on a well-established method for detecting lipid peroxidation [23]. The ability of anti-oxidative defense in response to increased oxidative damage was evaluated by measuring the serum level of total reduced thiols since serum thiols are physiologic free radical scavengers [24].

### Neuropsychological assessment of perceptual function

A clinical psychologist performed the neuro-psychological (NP) battery of tests from the Wechsler Adult Intelligence Scale-III (WAIS-III) [25]. The WAIS-III, with 14 subtests, allows us to summarize index scores and convert to different large ability areas reliably, including three IQs (Verbal IQ, Performance IQ, and Full Scale IQ) and four Indexes (Verbal Comprehension, Working Memory, Perceptual Organization, and Processing Speed).

In the current study, we focused on perceptual organization and processing speed functions in PD. These batteries comprise three tests for perceptual organization function and one test for processing speed function. The tests for perception organization function include picture completion, block design, and matrix reasoning tests. We sum the results from these tests to come up with an index score which represents the aggregate abilities of perceptual organization function.

The test for processing speed was a digit symbol-coding test which examines several cognitive processes of participants, such as detecting a test digit, searching for the corresponding symbol, and maintaining the symbol representation. We then further added scaled scores of perceptual organization and processing speed functions and converted them into the performance IQ.

### MR image acquisition

Volumetric structural MRI scans were acquired on a GE Signa 3T whole-body MRI scanner (General Electric Healthcare, Milwaukee, WI, USA) using an eight-channel phase array head coil at the Kaohsiung Chang Gung Memorial Hospital in Taiwan. Whole brain three-dimensional T1 weighted images were collected for each participant using an inversion-recovery fluid-attenuated fast spoiled gradient-recalled echo pulse sequence with the following imaging parameters: repetition time (TR)/echo time (TE)/inversion time (TI) = 9.5/3.9/450 ms; flip angle = 15 degrees; number of excitations (NEX) = 1; field of view (FOV) = 240 × 240 mm^2^; matrix size = 512 × 512; voxel size = 0.47 × 0.47 × 1.3 mm^3^; and slice number = 110 axial slices (without interslice gaps). In order to identify any brain abnormalities, an additional volumetric axial T2-weighted fast spin-echo sequence (TR/TE = 4200/102 ms; echo train length = 18; NEX = 2; FOV = 240 mm^2^; slice thickness = 5 mm; matrix size = 320 × 256 and 18 slices) and axial T2-weighted inversion-recovery fluid-attenuated sequence (TR/TE/TI = 8000/100/2000 ms; NEX = 1; FOV = 240 mm^2^; slice thickness = 5 mm; matrix size = 320*256 and 18 slices) were used in the same imaging session.

T1-weighted structural MRI scans were analyzed with voxel-based morphometry (VBM) [26] using the VBM8 toolbox (http://dbm.neuro.uni-jena.de), which was implemented using the Statistical Parametric Mapping software program (SPM8, Wellcome Institute of Neurology, University College London, UK, http://www.fil.ion.ucl.ac.uk/spm/) with default settings. All the image processing procedures, including tissue segmentation, study-specific template construction, and spatial normalization, were conducted according to a previous study [27] and are summarized in the Additional file 1 for the present study.

### Statistical analysis

#### Analysis of demographic data, neuropsychological assessments, and global tissue volumes

The demographic data, including age, sex, and education, were compared among the study groups by the 2-sample Student’s t test and Pearson’s Chi square test, where appropriate. Differences in the serum concentrations of TBARS and thiol, and in neuropsychological assessment scores and global tissue volumes, including the GM, WM, CSF volumes, and TIV, were analyzed using an analysis of covariance (ANCOVA) model with the participant’s age, sex, and education values as covariates. The threshold for all statistical significance was set at P < 0.05 (SPSS software, version 17, for Windows, Chicago, IL, USA).

#### Voxel-wised gray matter volume comparisons between healthy controls and patients with PD

To examine between-group differences in gray matter volume, we used a general linear model which was implemented in SPM8 to compare modulated gray matter segments between healthy controls and patients with PD at the level of the whole brain. All of the processes were conducted according to a previous study [27] and are summarized in the Additional file 1 for the present study. The resultant statistical inferences were considered significant under the criteria of cluster level family-wise error (FWE) corrected P value <0.05, with a cluster size of at least 184 voxels, based on the results of the Monte Carlo simulation (3dClusterSim with the following parameters: single voxel P value <0.005, FWHM = 7 mm with GM mask and 10,000 simulations).

#### Mediation analysis

**Fig. 1:**
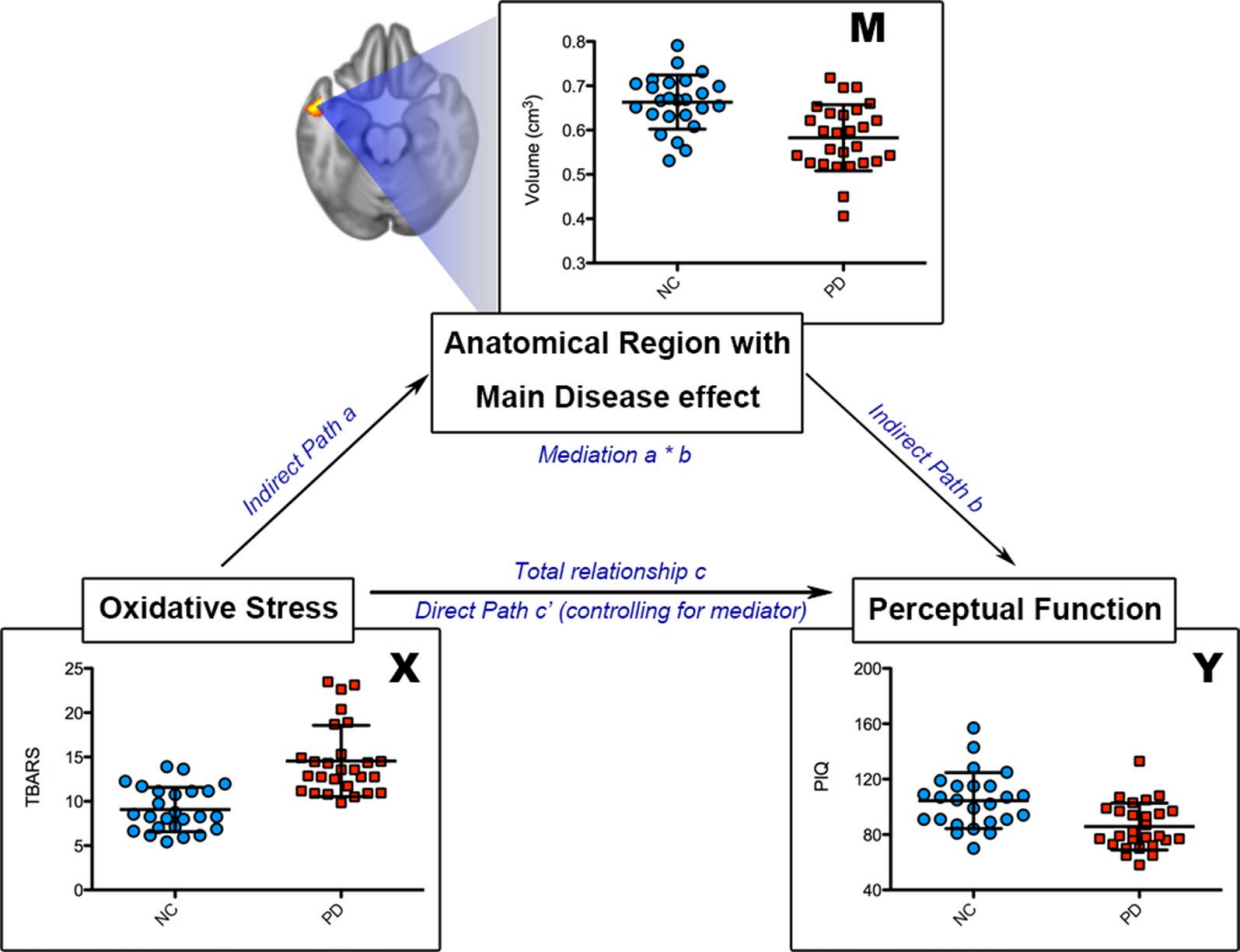
The diagram of the mediation hypothesis framework. In this mediation hypothesis framework, we want to identify the potential anatomical region which exhibited the mediation effect between oxidative stress level and perceptual function. We selected the oxidative stress as a predictor (*X*), perceptual functions as an outcome variable (*Y*), and regional gray matter with significant main disease effect (investigated from VBM analysis) as potential mediators (*M*). The three components of single level mediation analysis address the relationship between these variables. Indirect “path a” investigates the relationship between oxidative stress level and regional gray matter volume. Indirect “path b” investigates whether the regional gray matter volume predicts the perceptual functions after adjusting the oxidative stress level. Mediation a*b effect investigates whether regional gray matter volume plays the role of mediator between the oxidative stress level and perceptual functions. In addition, path c’ and c represent the total relationship between oxidative stress and perceptual functions with/without adjustments to regional gray matter volume, respectively. *PIQ* performance intelligence quotient, *TBARS* thiobarbituric acid reactive substances, *VBM* voxel-based morphometry

A single-level three-variable mediation model [28], illustrated in Fig. 1, was used to investigate the causal relationships between oxidative stress, regional GM volume, and perceptual function. Mediation analysis tests whether the direct effect of an independent variable on a dependent variable can be explained by the indirect influence of the mediating variable. The primary hypothesis of this analysis asks whether the effect of the oxidative stress level (independent variable) on the perceptual function (dependent variable) was explained indirectly by the regional gray matter volume changes (mediator) with significant group main effect. In order to examine this indirect effect, the Mediation toolbox (http://wagerlab.colorado.edu/tools) with an accelerated bias-corrected bootstrap test of statistical significance was used (10,000 bootstrap samples). The path model jointly tested three effects of interest that are required if a regional GM volume links oxidative stress with the perceptual function: (a) the effect of the independent variable (TBARS level) on the mediator (regional gray matter volume) (indirect effect, path a); (b) the effect of the mediator on the dependent variable (perceptual performances) by controlling the effect for the TBARS (indirect effect, path b); and (c) the mediation effect a × b which is defined as the reduction of the relationship between the independent and dependent variables (TBARS—perceptual performance) (total relationship, path c) by including the mediator into the model (direct path, path c′). We set the statistical significance threshold at 0.05 for all the relevant paths [29].

## Results

### Demographic data, perceptual scores, and serum concentrations of TBARS and thiol of PD patients and healthy controls

The demographic and clinical data of the participants are shown in Table 1. In the ANCOVA analyses, the patients with PD had worse performance on the perceptual function tests, including the picture completion (P = 0.007), block design (P = 0.002), matrix reasoning (P = 0.020), and digit symbol-coding tests (P < 0.001). Further analysis showed that the patients with PD had a worse mean score in terms of the perceptual organization index (P = 0.004). Moreover, when that mean was combined with the mean score for digital symbol-coding, the mean performance IQ in patients with PD was also found to be worse than the mean for the healthy controls (P = 0.002).

**Table 1:** Analysis of demographic variables, clinical variables, global anatomical measurements, and cognitive assessments between the healthy controls and patients with Parkinson’s disease

Variable	PD group (n = 27)	Control group (n = 25)	p value
Age (years)	54.59 ± 9.32	50.88 ± 10.51	0.183^a^
Sex (male/female)	11/16	11/14	1.000^b^
Education (years)	9.22 ± 4.91	11.76 ± 4.27	0.053^a^
GMV (l)	0.54 ± 0.06	0.57 ± 0.05	0.58^c^
WMV (l)	0.54 ± 0.05	0.57 ± 0.05	0.108^c^
CSFV (l)	0.22 ± 0.03	0.21 ± 0.03	0.412^c^
TIV (l)	1.29 ± 0.11	1.35 ± 0.11	0.032^d^*
UPDRS I	3.37 ± 2.53	–	–
UPDRS II	10.44 ± 8.06	–	–
UPDRS III	23.48 ± 12.64	–	–
UPDRS total score	37.15 ± 21.69	–	–
Modified HY-stage^e^	1.98 ± 1.05	–	–
SE-ADL^f^	85.19 ± 17.18	–	–
MMSE	25.04 ± 4.28		
PIQ	85.78 ± 16.93	104.52 ± 20.16	0.002^d^*
Perceptual organization index	85.74 ± 17.75	101.52 ± 17.38	0.004^d^*
Picture completion	7.33 ± 3.14	10.52 ± 4.44	0.007^d^*
Block design	7.07 ± 3.23	9.96 ± 2.88	0.002^d^*
Matrix reasoning	8.07 ± 3.82	10.60 ± 3.19	0.020^d^*
Processing speed index			
Digit symbol	6.81 ± 3.08	10.24 ± 2.30	<0.001^d^*
TBARS (μM)	14.5 ± 3.95	9.10 ± 2.50	<0.001*
Thiol (μM)	1.40 ± 0.50	1.50 ± 0.30	0.191

Means and standard deviations of raw scores for the healthy control group and the patients with Parkinson’s disease. For each variable, the p value indicates the significance level of the appropriate statistical test comparing the raw scores of the control group and the patients with Parkinson’s disease

*CSFV* cerebrospinal fluid volume, *GMV* gray matter volume, *MMSE* mini–mental state examination, *Modified H&Y stage* modified Hoehn and Yahr stages, *PD* Parkinson’s disease, *PIQ* performance intelligence quotient, *SE-ADL* Schwab and England activities of daily living scale, *TBARS* thiobarbituric acid reactive substances, *TIV* total intracranial volume, *UPDRS* Unified Parkinson’s Disease Rating Scale, *WMV* white matter volume

^a^Two sample unpaired *t* test

^b^Chi square test

^c^Analysis of covariance test which adjusted for age, sex, education, and TIV

^d^Analysis of covariance test which adjusted for age, sex, and education

^e^For Modified HY-stage, the maximum stage is 5

^f^For the SE-ADL, the minimum score is 0, suggesting vegetative functions; the maximum score is 100, suggesting completely independent

* P value < 0.05

The mean serum concentration of TBARS was significantly higher in the PD patients than in the controls (P < 0.001). The mean serum concentration of free thiol was lower in PD patients than in the controls, but the difference failed to achieve statistical significance (P = 0.191). Since only the serum TBARS level revealed a significant group difference, the following correlation and mediation analyses were primarily focused on and carried out with regard to the TBARS levels.

### Gray matter volume reduction in patients with Parkinson’s disease

**Fig. 2:**
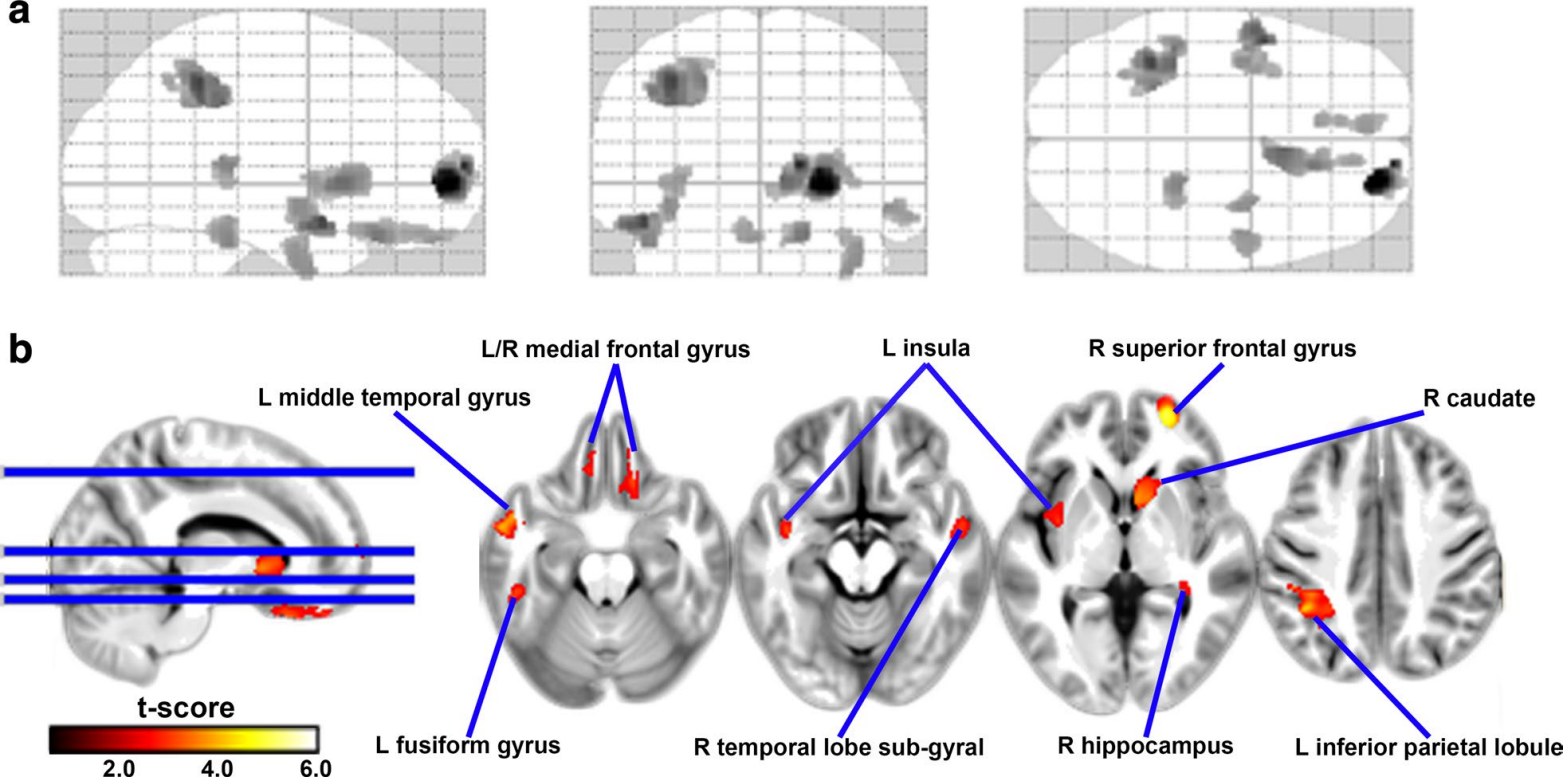
Reduced gray matter volume in PD using a whole brain voxel-wised exploratory analysis. Significant (cluster level statistics, p value <0.05, family-wise error corrected) regional *gray matter volume* reduction in a patient with Parkinson’s disease revealed by whole brain VBM analysis. **a** The spatial distribution of regional gray matter volume reduction. **b** VBM results are overlaid onto the mean group DARTEL-T1 template. The patient with Parkinson’s disease showed lower regional *gray matter volumes* in the left middle temporal gyrus, left fusiform gyrus, left insula, left inferior parietal lobule, right caudate, right hippocampus, right superior frontal gyrus, and bilateral medial frontal gyrus. The *hot color bar* indicates the T scores scale. *DARTEL* diffeomorphic anatomical registration through exponentiated lie algebra, *L* left, *R* right, *VBM* voxel-based morphometry

Compared to healthy controls, PD patients showed a significant GM volume reduction in the following brain regions: (1) bilateral temporal gyri (middle temporal gyrus and fusiform gyrus), (2) bilateral frontal gyri, (3) limbic lobe (hippocampus and uncus), (4) left inferior parietal lobule, (5) right caudate nucleus, and (6) insula. We did not find increased GM volume in PD patients compared with the healthy controls (Additional file 2: Table S2; Fig. 2).

### Correlations between serum TBARS and perceptual functions

Consistent with our hypotheses, elevated serum oxidative stress levels were associated with poorer performances on several perceptual subtests, which suggested that systemic inflammation might degrade general perceptual functions directly or indirectly in PD (Table 2). We found that higher TBARS levels were associated with worse matrix reasoning (p = 0.017, r = −0.337), digit symbol (p = 0.044, r = −0.286), and perceptual organization index (p = 0.035, r = −0.299) scores. We also found that high TBARS levels were borderline significantly correlated with worse block design (p = 0.066, r = −0.262), picture completion (p = 0.058, r = −0.270), and performance IQ (p = 0.062, r = −0.266) scores.

**Table 2:** Associations between oxidative stress level (TBARS) and perceptual functions after adjustments for age and sex

Clinical variable	Correlation coefficient	p value
Performance IQ	−0.266	0.062
Perceptual organization index	−0.299	0.035*
Picture completion	−0.270	0.058
Block design	−0.262	0.066
Matrix reasoning	−0.337	0.017*
Digit symbol	−0.286	0.044*

The results of partial correlation analysis between oxidative stress level and perceptual functions (adjusted for age, sex and education) were illustrated as correlation coefficients and corresponding p values

*TBARS* thiobarbituric acid reactive substances

* Indicates a P value of less than 0.05

### Brain mediators of oxidative stress on perceptual functions

#### TBARS-digit symbol relationship mediator

**Fig. 3:**
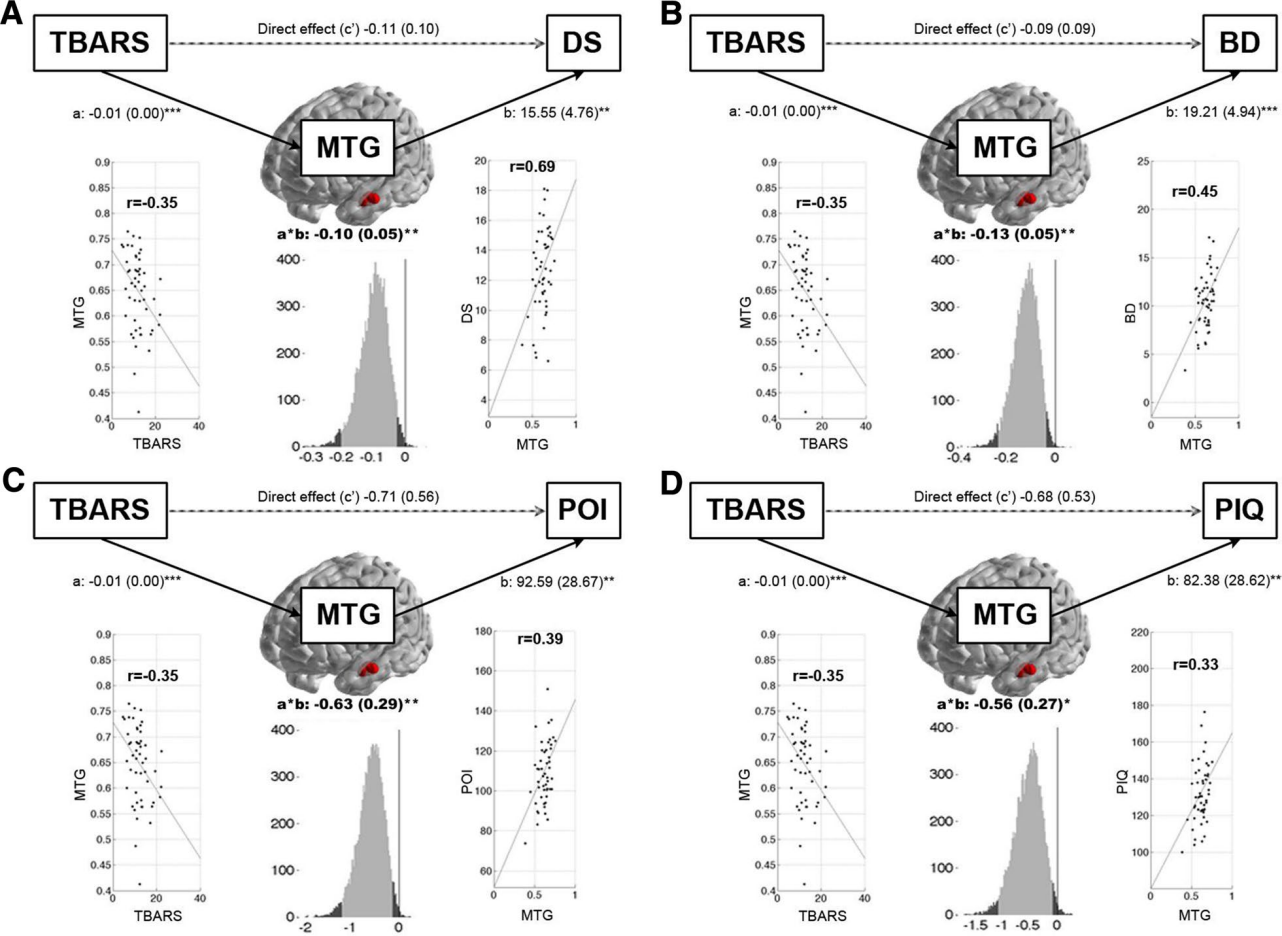
Middle temporal gyrus as potential brain mediator between oxidative stress level and different perceptual functions. Mediation path diagram showing the relationship between oxidative stress level (predictor, TBARS), regional *gray matter volume* (mediator, using MTG for visualization purpose, the other potential brain mediators and full statistics are presented in Table 3), and different perceptual functions (dependent variable, **A** DS; **B** BD; **C** POI; and **D** PIQ). The *dark solid* and *light dashed lines* indicate the significant/non-significant relationship between each variable, respectively. The hypothesized causalities are presented as *one-directional arrows*. The corresponding mean path coefficients with standard errors of the path a, path b, path c’, and mediation a*b effects for each mediation model are also labeled. The correlation scatterplots indicate the relationship between oxidative stress level, MTG *gray matter volume*, and different perceptual functions across whole study groups (“path a” and “path b” effects, respectively). The histogram shows the bootstrap estimates of mediation a*b effect distribution of MTG for each mediation model. The *light gray shading* shows the 95 % confidence intervals of bootstrap estimates and the *vertical line* indicates the null hypothesis value of zero. *p < 0.05, **p < 0.01, ***p < 0.001. *BD* block design, *DS* digit symbol, *MTG* middle temporal gyrus, *PIQ* performance intelligence quotient, *POI* perceptual organization index, *TBARS* thiobarbituric acid reactive substances

Single-level three-variable mediation analysis revealed atrophy of middle temporal gyrus that negatively mediated the TBARS-digit symbol relationship. The middle temporal gyrus volume reduction was negatively associated with TBARS levels, but predicted impaired digit symbol test performance (Coef_a_ = −0.007, P_a_ < 0.001; Coef_b_ = 15.55, P_b_ = 0.003; Coef_ab_ = −0.10, P_ab_ = 0.005). This finding suggests that the serum level of TBARS plays a role in contributing negative impact on digit symbol test scores through the middle temporal gyrus. Figure 3A shows path diagrams and scatterplots for path analyses in middle temporal gyrus. We also found that volume reductions in the inferior parietal lobule (Coef_ab_ = −0.10, P_ab_ = 0.042), hippocampus (Coef_ab_ = −0.06, P_ab_ = 0.042), and insula (Coef_ab_ = −0.12, P_ab_ = 0.025) can negatively mediate the TBARS-digit symbol relationship (Table 3).

**Table 3:** The potential brain mediators of the relationships between oxidative stress and the different perceptual functions

Clinical variable	Anatomy name	Path a			Path b			a × b				Path c’	
		p_coef_	z	p	p_coef_	z	p	p_coef_	z	p	p_coef_	z	p
Digit symbol	MTG	−0.007	−3.97	<0.001	15.55	2.94	0.003	−0.10	−2.82	0.005	−0.11	−2.12	0.034
	IPL	−0.009	−3.30	<0.001	11.29	2.28	0.023	−0.10	−2.04	0.042	−0.11	−2.20	0.028
	HIPP	−0.004	−2.49	0.013	18.25	−1.58	0.030	−0.06	−2.03	0.042	−0.15	−2.25	0.025
	INS	−0.005	−2.84	0.005	24.73	2.38	0.017	−0.12	−2.24	0.025	−0.09	−2.17	0.030
Block design	MTG	−0.007	−3.40	<0.001	19.21	4.02	<0.001	−0.13	−2.91	0.004	−0.09	−2.67	0.008
	HIPP	−0.004	−2.46	0.014	16.85	2.53	0.011	−0.06	−2.03	0.042	−0.15	−2.55	0.011
	INS	−0.005	2.86	0.004	29.32	3.03	0.002	−0.14	−2.47	0.014	−0.07	−2.56	0.011
POI	MTG	−0.007	−3.64	<0.001	92.59	2.84	0.005	−0.63	−2.74	0.006	−0.71	−2.72	0.007
	HIPP	−0.004	−2.44	0.015	99.80	2.42	0.015	−0.34	−2.15	0.031	−1.01	−2.71	0.007
	INS	−0.005	−2.99	0.003	142.70	2.88	0.004	−0.70	−2.39	0.017	−0.65	−2.75	0.006
PIQ	MTG	−0.007	−3.59	<0.001	82.38	2.60	0.009	−0.56	−2.26	0.024	−0.68	−2.62	−0.009
	INS	−0.005	−2.96	0.003	139.79	2.83	0.005	−0.68	−2.43	0.015	−0.57	−2.56	0.010

The corresponding statistical results of each path and mediation effect between the oxidative stress (independent variable), perceptual functions (dependent variable), and anatomical regions with significant disease effect (mediators) are described in terms of corresponding path coefficient, z value, and p value. The anatomical regions were considered as potential brain mediators between oxidative stress level and perceptual functions if statistical significance, as indicated by a p of less than 0.05, was found in each of the following three effects in corresponding mediation analysis. (1) Indirect Path a: the relationship between oxidative stress level and the regional gray matter volume; (2) indirect Path b: the relationship between the regional gray matter volume and perceptual functions as determined by controlling the oxidative stress level and (3) Mediation a × b effect. All mediation analyses were conducted using the Mediation toolbox

*P*_*coef*_ path coefficient, *HIPP* hippocampus, *INS* insula, *IPL* inferior parietal lobule, *MTG* middle temporal gyrus, *PIQ* performance intelligence quotient; POI, perceptual organization index

#### TBARS-block design relationship mediator

The middle temporal gyrus volume reduction was negatively associated with TBARS level, and predicted impaired block design test scores (Coef_a_ = −0.007, P_a_ < 0.001; Coef_b_ = 19.21, P_b_ < 0.001; Coef_ab_ = −0.13, P_ab_ = 0.004). This finding suggests that the serum level of TBARS also plays a role in contributing negative impact on block design test through the middle temporal gyrus (Fig. 3B). We also found that volume reductions in the hippocampus (Coef_ab_ = −0.06, P_ab_ = 0.042), and insula (Coef_ab_ = −0.14, P_ab_ = 0.014) can negatively mediate the TBARS-block design relationship (Table 3).

#### TBARS-perceptual organization index relationship mediator

Since the perceptual organization index is the sum of the picture completion, block design, and matrix reasoning scores, we also found that volume reductions in the middle temporal gyrus (Coef_ab_ = −0.63, P_ab_ = 0.006) (Fig. 3C), hippocampus (Coef_ab_ = −0.34, P_ab_ = 0.031), and insula (Coef_ab_ = −0.70, P_ab_ = 0.017) can negatively mediate the TBARS-perceptual organization index relationship (Table 3).

#### TBARS-performance intelligence quotient relationship mediator

Lastly, performance IQ is the sum of the picture completion, block design, matrix reasoning, and digit symbol-coding scores, and we also found that volume reductions in the middle temporal gyrus (Coef_ab_ = −0.56, P_ab_ = 0.024) (Fig. 3D) and insula (Coef_ab_ = −0.68, P_ab_ = 0.015) can negatively mediate the TBARS-performance IQ relationship (Table 3).

## Discussion

### Summary

Consistent with our hypothesis and in line with the extant literature, patients with PD experienced higher serum oxidative stress and worse perceptual functions. Furthermore, we identified gray matter atrophy throughout much of the cortex, including anatomical locations that typically appear to be particularly sensitive to the effects of inflammation, such as the temporal lobe, hippocampus, and insula [30]. We further demonstrated, for the first time, that gray matter volume atrophy in the temporal lobe, hippocampus, insula, and parietal lobe mediate the relationships between TBARS levels and general perceptual function scores. This suggests that higher oxidative stress levels are associated with poor perceptual functions by means of smaller temporal and mesolimbic volumes and that those regions support the retention of visual memories, the processing of sensory inputs, the comprehension of language, the storing of new memories and emotions in a manner that may differ from the way in which the prefrontal cortex supports executive functioning in PD.

### Pathophysiology of elevated oxidative stress in PD

Studies have demonstrated that an imbalance in pro-oxidant/antioxidant homeostasis can enhance the generation of toxic reactive oxygen species [31] and may be related to disease severity in PD [32]. The TBARS level is one of the markers of oxidative stress, and has been found to be elevated not only in the substantia nigra but also in the CSF and plasma in PD [33–35]. In addition, many cytokines, as well as TBARS, are also markers of reactive oxygen species (ROS) activity, which can worsen central neuron cell injury through the elevation of reactivated microglia in the substantia nigra [36]. Cytokines produced in the brain can freely diffuse past the blood–brain barrier (BBB), a phenomenon which has been demonstrated to induce memory impairments [37]. Our study supports the previous hypothesis that oxidative stress is implicated in the pathogenesis of PD.

In addition to the nigrostriatal region, inflammation activated microglial cells in PD were also observed in various brain regions, such as the hippocampus, temporal lobe, and other regions of the cerebral cortex [30]. Actually, increased oxidative stress/inflammation has also been found to be associated with temporal lobe damage in aging [38] due to atherosclerosis and Alzheimer’s disease [39], with the pathological accumulation of amyloid and neurofibrillary tangles. Two leading factors might contribute this temporal lobe vulnerability: relatively high receptor and messenger RNA expression for proinflammatory cytokines [40] in those regions and higher links to portions of the salience network, such as the insula, which has a role in regulating the immune system and can enhance the peripheral inflammatory conditions [41] and subsequently affect the neural organization of semantic memory [42]. From animal studies, it has been demonstrated that injections of inflammatory cytokines into the hippocampus and the overexpression of IL-1 messenger RNA in the hippocampus selectively deteriorate spatial and contextual memory processes [43]. In addition, elevated peripheral inflammation can stimulate IL-1 expression within the central nervous system, including the medial temporal lobe, and generates increased cytokine expression with subsequent impacts on temporal lobe-dependent memory [44]. The vagus nerve, which serves as a neurally mediated immune-brain pathway, can also enhance hippocampal activity by peripheral inflammatory challenge and electrical stimulation [45, 46]. Taken together, these data demonstrate the sensitivity of human medial temporal lobe structures to systemic oxidative stress/inflammation and provide mechanistic insights relevant to the broader literature linking severe or chronic inflammation to the attrition of human memory.

### Gray matter atrophy serves as a mediator of oxidative stress on perceptual functions

In a previous cortical thickness analysis of PD with mild cognitive impairment, patient groups revealed widespread cortical thinning compared with controls, including thinning of the right inferior temporal gyrus, left superior parietal cortex, precuneus, lateral occipital, temporal gyri anterior cingulate, and superior frontal gyri [1]. The same study also found that memory and visuospatial performance, the core feature of perceptual function, were associated with temporoparietal and superior frontal gyri thinning. Our results replicated these previous findings and also consistently demonstrated the interaction between perceptual functional impairment and volume atrophy in the middle temporal gyrus, inferior parietal lobe, hippocampus, and insula. Although fewer cortical regions were found to be involved in this study, the relatively small sample size and younger patient group might have led to these slightly different results. Furthermore, different gray matter quantitative morphometrical evaluation methods which are sensitive to different properties of gray matter tissue (i.e., volume vs. cortical thickness) might also have led to the different outcomes. We do believe, however, that the results of the current study, together with those of the previous investigation, indicate that particular cortical atrophy can serve as a marker for perceptual function decline in early PD.

The test of mediation amounts to a test of whether controlling for each brain mediator explains a significant amount of the covariance between the independent variable and the dependent variable. In the initial analyses, we found that higher serum TBARS levels were associated with worse perceptual functions. We further examined whether the relationship between serum TBARS levels and perceptual functions was explained indirectly by greater damage to perceptual functional specific anatomies in patients with PD using mediation analyses. We found that the middle temporal lobe and insula, as well as the hippocampus, play the central roles in mediating most of the TBARS-perceptual function relationships. Interestingly, the caudate nucleus and frontal lobe volume reduction, which is a hallmark of PD, failed to mediate the association between oxidative stress and perceptual function. Actually, perceptual impairment in PD occurs not only on visuomotor tasks requiring a complex motor response such as drawing but also on visuoperceptual tasks that require a limited motor response. Furthermore, this perceptual impairment is not related to a decrement in intellectual abilities and can be clearly distinguished from dementia [4]. In the present study, though the results could not exclude the influence of TBARS on other aspects of systemic status, they suggest that TBARS may play a particular role in the perceptual deficits observed in PD. Another explanation might be the ceiling effect, which suggests that inflammation occurs earlier in the striatum and then is followed by inflammation in the temporal lobe and hippocampus, resulting in the sequential clinical presentations progressing from movement disorders to cognitive impairments. These results, however, should be interpreted with caution.

The interpretation of the findings here must be tempered by some of the limitations of the present study. We do not know the temporal relationships between peripheral inflammation, symptomatology, and anatomical integrity. Further task design or longitudinal evaluation with manually controlled systemic oxidative stress levels in animal models should be carried out in the future. In addition, the measurement of only a few biomarkers of oxidative damage cannot be considered a valid tool for exploring the multifaceted, complex oxidant/antioxidant imbalance in PD. Furthermore, the oxidant/antioxidant balance of PD patients may be influenced by a multitude of parameters. The interactions between the CNS and systemic oxidative stress and the effect of individual genetic variations and physical exercise could also affect either the systemic oxidative stress or the brain volume, and these interactions were not well evaluated in the present study. While systemic oxidative stress is also likely to have widespread effects on the brain tissue, the specific anatomical volumes that mediate a given cognitive function are likely to differ depending on the examinations being assessed [47]. In addition, recent research suggests that network-based rather than regional anatomic involvements contribute to the neurodegeneration and behavioral differences seen in PD, such that assessing structural or functional connectivity between brain regions associated with these phenotypes would clarify the complex neural network involved in oxidative stress–cognition relationships.

## Conclusions

Although extensive structural alterations might occur in PD, our results highlight the possibility that only some vulnerable anatomies, such as the temporal lobe and hippocampus, might mediate the systemic oxidative stress seen in certain phenotypes of PD. This model has important implications for understanding how systemic oxidative stress may result in the development of cognitive malfunctions in PD and might provide novel targets for candidate neuroprotective therapies.

## Additional files

10.1186/s12967-015-0749-9

10.1186/s12967-015-0749-9 Brain regions with gray matter volume reduction in the patients with Parkinson’s disease compared with the healthy control group.

## Abbreviations

ANCOVA: analyzed by analysis of covariance
HY-stage: Hoehn and Yahr stages
MRI: magnetic resonance imaging
IQ: intelligence quotient
PD: Parkinson’s disease
SE-ADL: Schwab and England activities of daily living scale
TBARS: thiobarbituric acid reactive substances
UPDRS: Unified Parkinson’s Disease Rating Scale
VBM: voxel-based morphometry
WAIS: Wechsler Adult Intelligence Scale-III
