# Supplementary material for: Brain mediators of systemic oxidative stress on perceptual impairments in Parkinson’s disease
Source: J Transl Med. 2015 Dec 21;13:386. doi: 10.1186/s12967-015-0749-9 (PMC4687285; doi:10.1186/s12967-015-0749-9)
Supplement: Supplementary file 1 — 10.1186/s12967-015-0749-9 [file 12967_2015_749_MOESM1_ESM.doc]

**The processing pipeline of DARTEL T1 voxel-based morphometry**

First, all native space T1-weighted structural MRI scans were bias corrected and segmented into gray matter (GM), white matter (WM), and cerebrospinal fluid (CSF) components using the tissue-prior free segmentation approach available in the VBM8 toolbox. The VBM8 segmentation included partial volume estimation to account for voxels mixed with two tissue types.[^1^](#_ENREF_1) The algorithm uses an adaptive Maximum A Posterior (MAP) approach which does not rely on a priori information regarding tissue probabilities[^2^](#_ENREF_2) and a subsequent application of a hidden Markov random field model[^3^](#_ENREF_3) to account for intensity inhomogeneities and variations without needing the tissue priors during tissue segmentation. This segmentation approach avoided introducing systematic bias into the segmentation procedure, which used the standard adult reference data as priori knowledge. To remove the global brain volume differences across subjects while retaining large-scale, inter-individual morphometric variation, these tissue segments were affine registered to the Montreal Neurological Institute (MNI) standard space tissue probability maps. After visually checking the affine-registered tissue segments for segmentation errors, the GM and WM segments of all participants were then used to create a study speciﬁc template using the diffeomorphic anatomical registration through exponentiated lie algebra (DARTEL)[^4^](#_ENREF_4) toolbox in SPM8. The resulting GM tissue segments were modulated by non-linear transformations derived from the DARTEL registration procedure and interpolated to an isotropic voxel size of 1.5 mm.[^5-7^](#_ENREF_5) This modulation procedure allowed us to make inferences based on relative local volumes (controlling for overall brain size) rather than on tissue concentrations for the subsequent statistical analysis of group comparisons and mediation analysis. Following the above procedure, we also performed a further quality check routine, which was implemented in the VBM8 toolbox, using covariance-based inhomogeneity measures of the resulting GM tissue segments. Finally, the resulting MNI space modulated GM segments were smoothed using the Gaussian kernel with full-width at half maximum in 8 mm. GM, WM, CSF volume and total intracranial volume (TIV) were estimated in cubic millimeters in the native space.

**Voxel-wised gray matter volume comparisons between healthy controls and patients with PD**

To examine between-group differences in gray matter volume, we used a general linear model which was implemented in SPM8 to compare modulated gray matter segments between healthy controls and patients with PD at the level of the whole brain. A one-factor 2-level ANCOVA design was utilized to reveal where healthy controls exhibited greater GM volumes than patients with PD and vice versa. To remove confounding effects of variables known to influence the results of VBM analysis, age, sex, and education were entered as nuisance variables into the ANCOVA model. To further avoid possible partial volume effects around the boundary between GM and WM, all voxels with a GM probability lower than 0.2 were eliminated in the statistical analysis. The resultant statistical inferences were considered significant under the criteria of cluster level family-wise error (FWE) corrected P value < .05, with a cluster size of at least 184 voxels, based on the results of the Monte Carlo simulation (3dClusterSim with the following parameters: single voxel P value < .005, FWHM = 7 mm with GM mask and 10000 simulations). The statistical result for clusters with significant between-group main effect, including cluster size, maximum Z-score, corresponding location of MNI coordinates, and anatomical structure, were reported using the Peak_nii toolbox (<http://www.nitrc.org/projects/peak_nii>). The mean volume of the clusters with significant group main effect was extracted for further mediation analysis.

1. Tohka J, Zijdenbos A, Evans A. Fast and robust parameter estimation for statistical partial volume models in brain MRI. Neuroimage 2004;23(1):84-97.

2. Rajapakse JC, Giedd JN, Rapoport JL. Statistical approach to segmentation of single-channel cerebral MR images. IEEE transactions on medical imaging 1997;16(2):176-186.

3. Cuadra MB, Cammoun L, Butz T, Cuisenaire O, Thiran JP. Comparison and validation of tissue modelization and statistical classification methods in T1-weighted MR brain images. IEEE Trans Med Imaging 2005;24(12):1548-1565.

4. Ashburner J, Friston KJ. Voxel-based morphometry--the methods. Neuroimage 2000;11(6 Pt 1):805-821.

5. Bezzola L, Merillat S, Gaser C, Jancke L. Training-induced neural plasticity in golf novices. J Neurosci 2011;31(35):12444-12448.

6. Muhlau M, Winkelmann J, Rujescu D, et al. Variation within the Huntington's disease gene influences normal brain structure. PLoS One 2012;7(1):e29809.

7. Ziegler G, Dahnke R, Jancke L, Yotter RA, May A, Gaser C. Brain structural trajectories over the adult lifespan. Human brain mapping 2012;33(10):2377-2389.
