# Supplementary material for: Brain mediators of systemic oxidative stress on perceptual impairments in Parkinson’s disease
Source: J Transl Med. 2015 Dec 21;13:386. doi: 10.1186/s12967-015-0749-9 (PMC4687285; doi:10.1186/s12967-015-0749-9)
Supplement: Supplementary file 2 — 10.1186/s12967-015-0749-9 Brain regions with gray matter volume reduction in the patients with Parkinson’s disease compared with the healthy control group. [file 12967_2015_749_MOESM2_ESM.docx]

**Supplementary table. Brain regions with gray matter volume reduction in the patients with Parkinson’s disease compared with the healthy control group**

| **MNI Coordinates** | | | **Cluster size** | **Side** | **Anatomical Region** | **t-score** |
| --- | --- | --- | --- | --- | --- | --- |
| **x** | **y** | **z** |  |  |  |  |
| 23 | 54 | -3 | 581 | Rt. | Superior Frontal Gyrus | 5.31 |
| -36 | -44 | 45 | 1148 | Lt. | Inferior Parietal Lobule | 4.51 |
| -52 | 2 | -22 | 219 | Lt. | Middle Temporal Gyrus | 4.45 |
| 8 | 11 | -2 | 538 | Rt. | Caudate | 4.02 |
| 15 | 26 | -26 | 290 | Rt. | Inferior Frontal Gyrus | 3.78 |
| 24 | -35 | 6 | 195 | Rt. | Hippocampus | 3.55 |
| -48 | -35 | -27 | 212 | Lt. | Fusiform Gyrus | 3.52 |
| -39 | -6 | -12 | 381 | Lt. | Insula | 3.41 |
| 36 | -6 | -35 | 204 | Rt. | Uncus | 3.37 |
| 50 | -6 | -14 | 199 | Rt. | Temporal Lobe Sub-Gyral | 3.32 |
| -6 | 50 | -27 | 237 | Lt. | Medial Frontal Gyrus | 3.28 |

Gray matter volume reductions in patients with Parkinson’s disease are described in terms of MNI coordinates, cluster extent, brain side, and corresponding anatomical regions. The T-score of the voxel with the strongest group effect in a given cluster is also listed. The statistical criteria of the VBM results were set as a cluster level FWE corrected P value < 0.05 (using a Monte Carlo simulation to correct the multiple comparison problem). Abbreviations: FWE, family wised error; Lt, left side; MNI, Montreal Neurological Institute; Rt, right side; VBM, voxel-based morphometry
